# Development of multi-algorithm machine learning models integrating novel serum biomarkers for survival prediction in colorectal cancer: a retrospective cohort study

**DOI:** 10.3389/fonc.2026.1904591

**Published:** 2026-09-15

**Authors:** Hailun Xie, Lishuang Wei, Taiqi Chen, Shuangyi Tang, Jialiang Gan

**Affiliations:** 1 Department of Gastrointestinal and Gland Surgery, Nanning, Guangxi, China; 2 Guangxi Key Laboratory of Enhanced Recovery After Surgery for Gastrointestinal Cancer, The First Affiliated Hospital, Guangxi Medical University, Nanning, Guangxi, China; 3 Department of Colorectal and Anal Surgery, The First Affiliated Hospital, Guangxi Medical University, Nanning, Guangxi, China; 4 Department of Pharmacy, The First Affiliated Hospital, Guangxi Medical University, Nanning, Guangxi, China

**Keywords:** colorectal cancer, machine learning, nomograms, overall survival, progression-free survival

## Abstract

**Background:**

Colorectal cancer (CRC) prognosis prediction remains challenging due to tumor heterogeneity, and existing machine learning-based prognostic studies often rely on single algorithms or limited feature types. This study aims to employ a multi-algorithm collaborative strategy to identify critical prognostic factors for CRC patients and develop integrated prognostic nomograms that combine multi-dimensional features.

**Methods:**

Deep machine learning analysis was conducted in the R environment using the randomForest, glmnet (for LASSO regression), and caret packages. Three algorithms (LASSO regression, XGBoost, Random Forest) were applied to screen 73 clinicopathological features, with key hub features identified as the intersection of results from the three methods. Survival analysis was performed using the Kaplan-Meier method (log-rank test), and Cox regression analysis confirmed independent prognostic associations. Nomogram performance was validated via calibration curves, time-dependent AUC, C-index, decision curve analysis (DCA), and internal validation cohorts.

**Results:**

The multi-algorithm intersection identified 6 key hub features encompassing pathological (M stage, N stage), serological (Cystatin C, Homocysteine, γ-Glutamyl Transferase), and demographic (age) factors. Cox regression confirmed these as independent predictors of overall survival (OS) and progression-free survival (PFS) in CRC patients. Prognostic nomograms developed for 1–5 year OS and PFS showed excellent calibration (consistent predicted vs. observed survival) and high accuracy (OS C-index: 0.730, 95% CI: 0.707-0.753; PFS C-index: 0.722, 95% CI: 0.700-0.744; AUCs >75% for OS, >77% for PFS). DCA demonstrated superior clinical utility of the nomograms over traditional pathological staging. Internal validation cohorts confirmed robust predictive performance.

**Conclusions:**

Our study innovatively uses a multi-algorithm cross-validation strategy to identify 6 multi-dimensional hub features. It is the first to comprehensively integrate clinical, pathological, serological, and demographic factors into CRC prognostic nomograms, which outperform traditional staging and enable more personalized, clinically actionable prognosis prediction for CRC patients.

## Introduction

Colorectal cancer (CRC) is a global health issue with high incidence and mortality rates worldwide. According to the latest global cancer statistics report, CRC ranks third in incidence among all cancer types in 2022, with 1,926,118 new cases (accounting for 7.3%). Simultaneously, the mortality rate of CRC ranks second among all cancer types, with 903,859 new deaths (accounting for 9.3%) (1). In China, the burden of CRC is also significant, with incidence ranking second and mortality ranking fourth (2). Over the past decades, overall survival rates have improved, mainly due to advancements in surgical techniques, radiotherapy, and chemotherapy, as well as the expansion and improved efficacy of surgical indications for metastatic disease (3–5). However, colorectal cancer treatment still faces many challenges. Although new treatments such as immunotherapy and targeted therapy have emerged, there are still issues. For instance, some patients may develop resistance to drugs, leading to treatment failure. Additionally, the high recurrence rate of CRC results in unsatisfactory long-term survival rates. Conventional TNM pathological staging, the mainstream prognostic stratification tool in clinical practice, only relies on anatomical tumor spread status and ignores individual systemic metabolic differences, which cannot fully explain the substantial survival heterogeneity observed among patients within identical TNM stages. This critical limitation creates an urgent clinical demand for multi-dimensional prognostic assessment systems integrating both pathological indicators and circulating laboratory markers. Therefore, it is crucial to find effective prognostic tools to accurately predict the prognosis of CRC patients and plan individualized treatment. Only in this way can we provide better treatment and quality of life for patients.

In previous studies, some tumor pathology-related prognostic factors of CRC patients have been reported, including staging and metastatic status (6–9). However, due to the heterogeneity of tumors, there are significant differences in treatment outcomes and responses even among patients at the same disease stage. Recently, easily accessible and non-invasive serum biomarkers have gained attention in predicting the prognosis of tumor patients and guiding treatment. Therefore, in addition to pathological factors, it is necessary to identify simple and effective serological prognostic markers to help further identify high-risk patients with poor prognosis. Moreover, the accuracy of predicting the prognosis of CRC patients using a single factor is not high, and combining multiple factors to comprehensively predict patient prognosis has important clinical value.

The three serum markers adopted in the present study—cystatin C, homocysteine, and γ-glutamyl transferase—are routinely measured items in clinical biochemical tests, yet their combined prognostic value for CRC remains underexplored. Cystatin C is a sensitive marker of renal function that correlates with systemic sarcopenia and tumor invasive capacity, reflecting overall metabolic dysfunction that impairs anti-tumor treatment tolerance. Elevated homocysteine drives chronic systemic inflammation and endothelial vascular damage, which facilitates the formation of pre-metastatic niches and increases CRC recurrence risk. γ-glutamyl transferase, a liver function enzyme, serves as a surrogate marker for occult hepatic metastasis and predicts inferior therapeutic response to systemic anti-tumor regimens. Despite their independent prognostic associations validated by prior independent cohorts, few existing predictive models integrate these three circulating indicators together with TNM staging parameters to build a comprehensive risk stratification tool for surgical CRC patients, leaving an important research gap.

Machine learning, as a new type of artificial intelligence technology, is beginning to be widely used in healthcare data analysis. By leveraging the powerful predictive capabilities of machine learning algorithms, it is possible to develop predictive tools superior to traditional statistical modeling, thereby better predicting the adverse prognosis of CRC patients. However, the benefits of machine learning in CRC risk assessment have not been fully explored. Therefore, this study aims to rank the importance of common clinical pathological factors using different machine learning methods to identify the most important risk factors for the prognosis of CRC patients. Additionally, we constructed the nomograms of these clinical pathological factors and performed internal validation to provide treatment recommendations for patients at varying risk levels and to assist in clinical decision-making.

## Materials and methods

### Study population

We retrospectively retrieved data from inpatients who underwent surgery in the Department of Colorectal and Anal Surgery at the First Affiliated Hospital of Guangxi Medical University from 2012 to 2015. Inclusion criteria were patients aged 18 years and older with pathologically confirmed colorectal adenocarcinoma and complete clinical pathological data. For M1-stage metastatic patients, only those diagnosed with oligometastatic disease and receiving curative-intent simultaneous resection of both the primary colorectal tumor and limited distant metastatic lesions were included in the cohort. Exclusion criteria included: 1) presence of other types of primary tumors; 2) prior chemotherapy or radiotherapy for colorectal cancer before surgery; 3) incomplete follow-up data; 4) M1 patients with extensive, unresectable distant metastases who only underwent palliative primary tumor resection to relieve clinical symptoms without radical metastasectomy. This retrospective study was approved by the Ethics Committee of the First Affiliated Hospital of Guangxi Medical University, with all patients signing written informed consent.

### Data collection

A total of 73 demographic and clinical data items were collected at enrollment. Demographic information included gender, age, height, weight, and comorbidities (hypertension and diabetes). Disease-related data included pT stage, pN stage, metastasis (distant metastasis), TNM stage (8th edition of the American Joint Committee on Cancer standards), tumor primary site (rectum, colon), tumor size (diameter <5 cm, ≥5 cm), neural/vascular invasion, pathological type, and differentiation degree. All hematological, biochemical and metabolic laboratory parameters were obtained after patient admission and prior to surgical resection, including complete blood count, liver function, kidney function, and tumor markers. The complete list and categorization of the 73 demographic and clinical data items are provided in **Supplementary Table 1**.

By collecting outpatient medical records or directly contacting patients or their relatives via telephone, we recorded the survival status (alive/dead) and recurrence status (yes/no) of patients. The follow-up procedure was every 3 months for the first 2 years after surgery, then every 6 months thereafter, with the last follow-up date in July 2023. Our study enrolled a total of 1329 patients. At the end of follow-up, 518 patients died, 745 patients were still alive, and 66 patients were lost to follow-up. The overall loss-to-follow-up rate was about 5.0%. Progression-free survival (PFS) was defined as the interval from radical tumor resection to the first progression, death, or the last follow-up date. Overall survival (OS) was defined as the interval from radical tumor resection to death or the last follow-up date.

### Multiple machine learning algorithms

To screen important clinical pathological features for predicting the prognosis of CRC, we analyzed 73 clinical features using Least absolute shrinkage and selection operator (LASSO) regression, Extreme Gradient Boosting (XGBoost) algorithm, and Random Forest algorithm (RF). Hyperparameters of LASSO, XGBoost and Random Forest were tuned by 10-fold cross-validation based on the caret package to obtain optimal model parameters and reduce overfitting risk. We then took the intersection of key clinical pathological features selected by LASSO regression, XGBoost algorithm, and RF as important features for predicting the prognosis of CRC patients. After identifying these consistent hub features via three machine learning algorithms for feature screening, we constructed clinically applicable prognostic nomograms for CRC patients using multivariate Cox proportional hazards regression.

### Statistical analysis

This study used R software (version 3.6.1) for statistical analysis. A p-value of less than 0.05 (two-sided) was deemed statistically significant. Categorical variables were presented as frequencies and proportions, and comparisons were conducted using Pearson’s chi-square test. The Shapiro-Wilk test was used to assess the normality of quantitative data. Non-normally distributed data were presented as medians with interquartile ranges (IQR). Comparisons of normally distributed quantitative parameters were performed using Student’s t-test, while comparisons of non-normally distributed quantitative parameters were made using the Mann-Whitney U test. Deep machine learning analysis was conducted in the R environment using the randomForest, glmnet, and caret packages. Survival analysis was performed using the Kaplan-Meier method, with survival differences compared by the log-rank test. Cox regression analysis was used to determine the independent association of significant features with prognosis. In the R environment, a prognostic nomogram for CRC patients including significant features was plotted based on the ‘regplot’ package. Additionally, time-dependent receiver operating characteristic (ROC) curves at 1 year, 3 years, and 5 years were plotted, and time-dependent area under the curve (AUC) values were calculated to assess the discriminative ability of the nomogram. Calibration curves were established to show the calibration of the nomogram, and the C-index was calculated to evaluate predictive accuracy. To demonstrate that the clinical benefit of the nomogram was superior to traditional TNM staging, decision curve analysis (DCA) was performed. For internal validation, all enrolled patients were randomly split into two equal subgroups at a 1:1 ratio. Calibration curves, time-dependent ROC curves, C-index and DCA were adopted to evaluate the predictive performance of the nomogram in both validation cohorts.

## Results

### Demographic characteristics

**Supplementary Table 2** summarizes the clinicopathological characteristics of 1263 CRC patients. The retrospective study cohort comprised 799 males (63.3%) and 464 females (36.7%), with an average age of 59.23 ± 12.63 years. There were 252 patients (20.0%) in stage I, 427 patients (33.8%) in stage II, 473 patients (37.5%) in stage III, and 111 patients (8.8%) in stage IV. The median follow-up time was 64.7 months. During the follow-up period, 581 patients died, and 398 patients experienced recurrence and metastasis. Patients who died typically had lower BMI, later staging, were more prone to perineural invasion, vascular invasion, low differentiation, higher CEA levels, and higher recurrence rates. The hospitalization duration for deceased patients was 4 days longer than for surviving patients; the hospitalization cost for deceased patients was over 2900 RMB higher than for surviving patients (**Supplementary Table 3**).

### Identification of important clinicopathological characteristics via multi-machine learning methods

To identify important clinical pathological features, we employed diverse machine learning algorithms to rank the importance of 73 clinical pathological features. In the LASSO regression, we observed 29 clinical pathological features with the minimum binomial deviation on the curve when the optimal lambda value was 0.022 (**Figures 1A, B**). **Figure 1C** shows the importance ranking of these features, where M stage, N stage, Cystatin C, CEA, and vascular invasion ranked high. Next, we applied the XGBoost algorithm to select the top 15 features based on importance scores (**Figures 2A, B**). In the RF algorithm, the top 12 clinical pathological features predicted poor prognosis for CRC patients with the lowest error and highest accuracy (**Figures 3A, B**). Although the importance of variables varied slightly among different machine learning algorithms, the pathological features of the tumor remained at the forefront. Finally, we took the intersection of important clinicopathological characteristics obtained from the three machine learning algorithms and visualized it through a Venn diagram, resulting in 6 candidate hub clinicopathological features: M stage, N stage, Cystatin C, Homocysteine, Y-Glutamyl Transferase, and Age (**Figure 4**).

**Figure 1:**
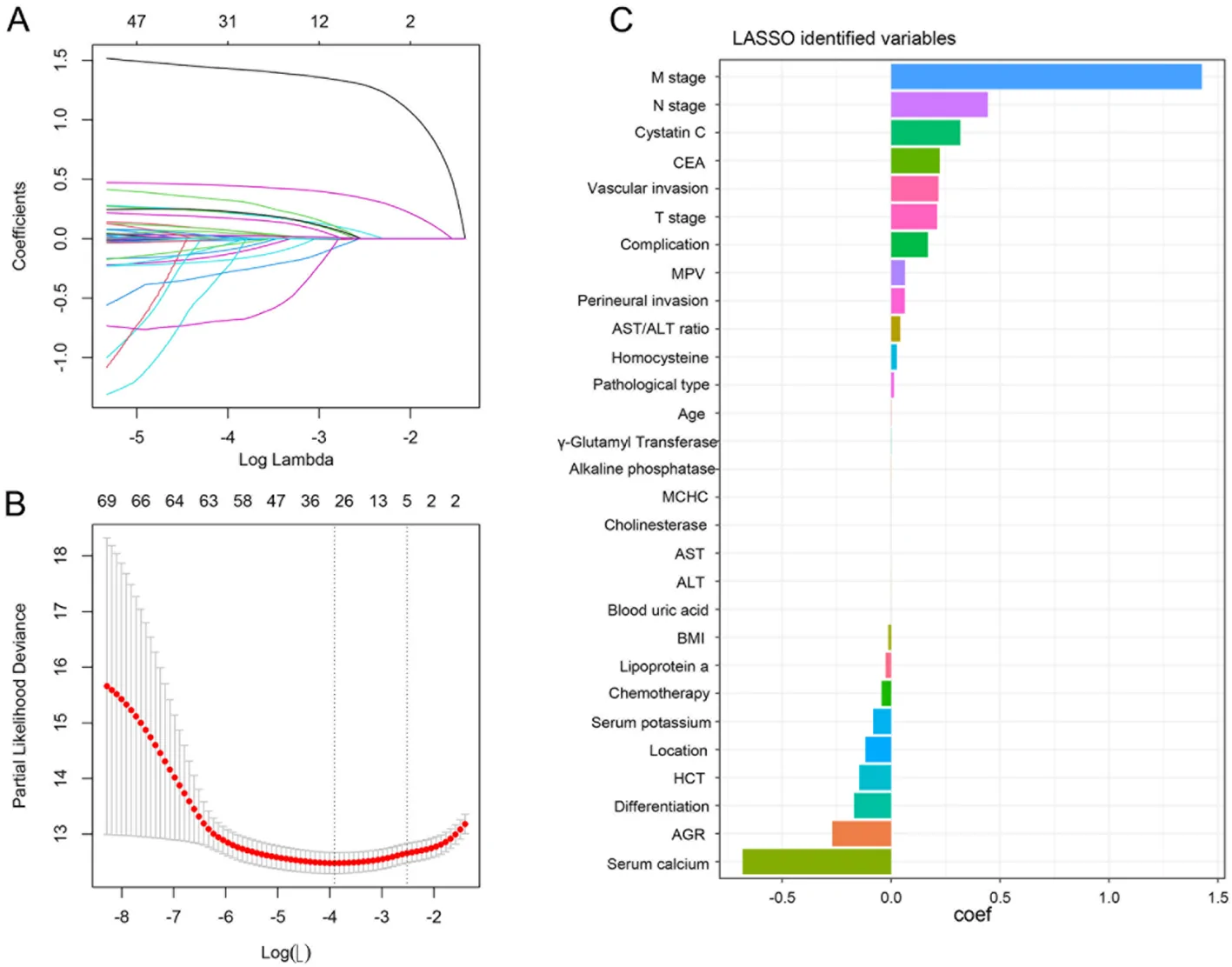
Feature selection for CRC prognostic factors using LASSO regression. This image presents the process of screening prognostic features from 73 initial clinicopathological variables via LASSO regression, aiming to reduce dimensionality and exclude noisy variables for subsequent multi-algorithm validation. **(A)** The cross-validation (cv) curve was plotted to determine the optimal lambda (λ) value: the vertical dashed line indicates λ = 0.022, which minimized the binomial deviation and balanced model complexity and predictive accuracy. **(B)** Results of 10-fold cross-validation: the x-axis represents log(λ), and the y-axis represents the regression coefficient of each variable; as λ increased, variables with weaker predictive power were gradually eliminated, ultimately retaining 29 candidate features. **(C)** Ranking of variables identified by LASSO regression: the x-axis shows the regression coefficient of each retained feature, reflecting its association strength with CRC prognosis; M stage, N stage, Cystatin C, CEA, and vascular invasion ranked among the top, indicating their potential as key prognostic factors.

**Figure 2:**
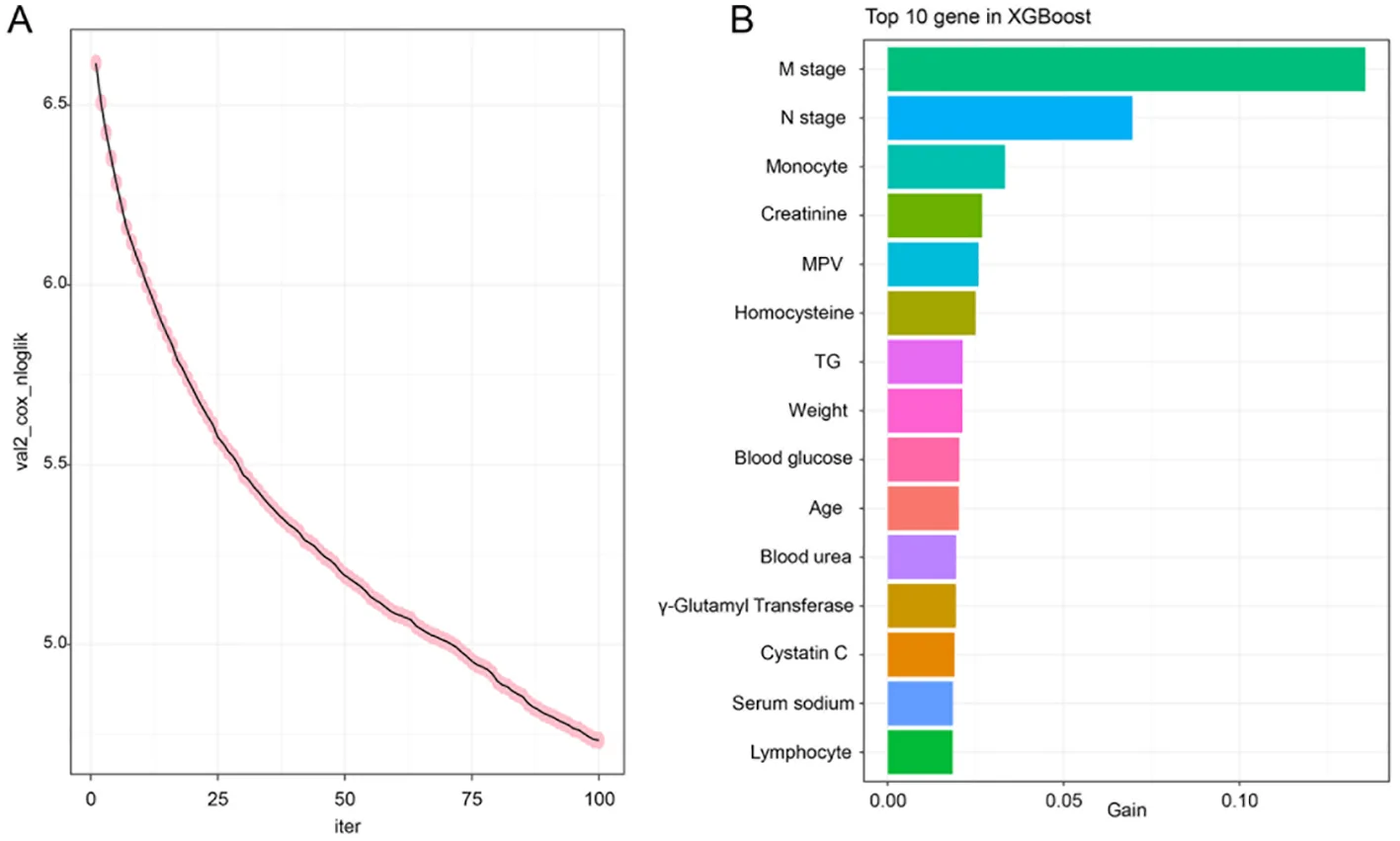
Feature selection for CRC prognostic factors using the XGBoost algorithm. This image illustrates the XGBoost-based screening of 73 clinicopathological features to identify variables with high prognostic importance, complementing LASSO regression’s linear feature selection with nonlinear relationship mining. **(A)** The negative partial log-likelihood of Cox proportional hazards regression in the XGBoost model is plotted against the number of iterations: the curve stabilized as iterations increased, indicating the model converged (no further reduction in prediction error), confirming the reliability of feature importance scores. **(B)** Ranking of the top 15 features by importance score (x-axis): M stage and N stage had the highest scores, followed by monocyte count, creatinine, MPV, HCY, age, and GGT; these features were prioritized for subsequent intersection analysis with other algorithms.

**Figure 3:**
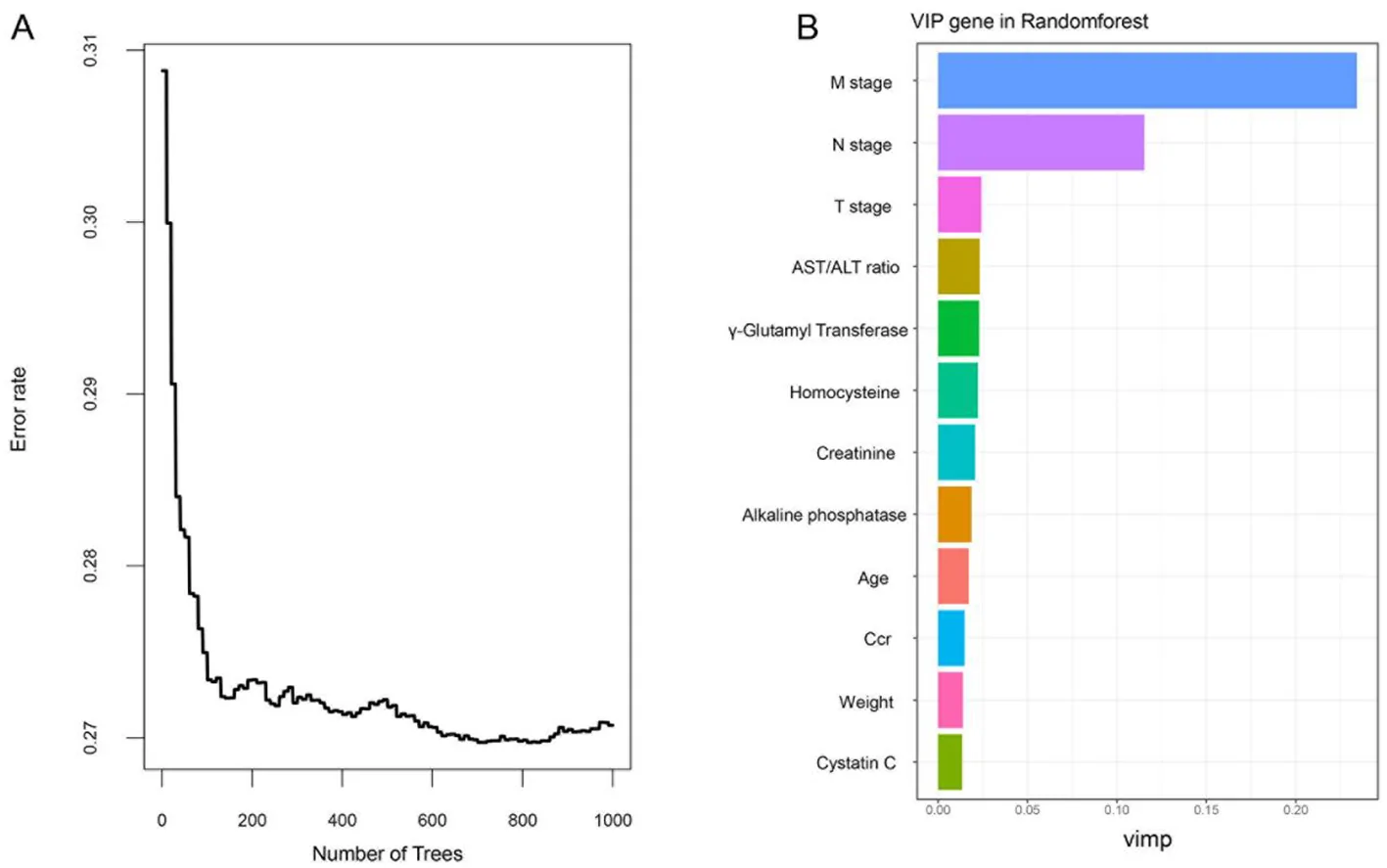
Feature selection for CRC prognostic factors using the random forest algorithm. This image describes the RF-based evaluation of 73 clinicopathological features, leveraging decision-tree ensemble learning to capture complex nonlinear associations between variables and CRC prognosis. **(A)** Out-of-Bag (OOB) error plot: the x-axis represents the number of decision trees, and the y-axis represents OOB error (a measure of model generalization); the error stabilized at ~0.27–0.29 when the number of trees exceeded 600, indicating the model was sufficiently stable to identify 12 key prognostic features. **(B)** Ranking of variables by Variable Importance (VIP) score (x-axis): M stage, N stage, T stage, AST/ALT ratio, GGT, HCY, creatinine, and age were among the top VIP features, consistent with trends from LASSO and XGBoost, supporting their potential as robust prognostic factors.

**Figure 4:**
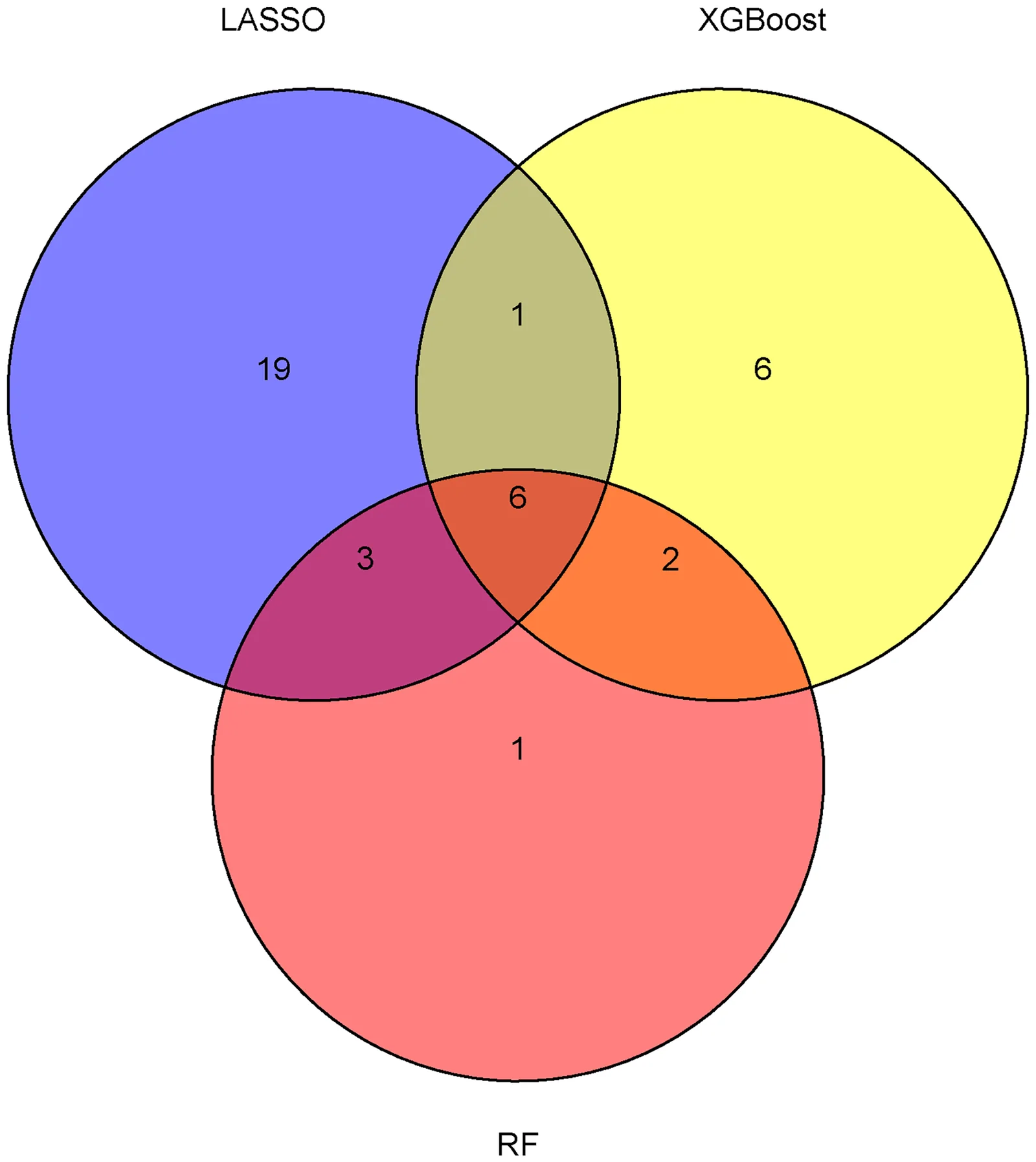
Venn diagram of intersection features identified by three machine learning algorithms. This Venn diagram visualizes the overlap of candidate prognostic features screened by LASSO regression, XGBoost, and RF, to ensure the final hub features are robust across different algorithmic frameworks. The three circles represent the feature sets from each algorithm: LASSO regression retained 29 features, XGBoost identified 15 top features, and RF selected 12 VIP features. The overlapping region (6 features) represents the final hub prognostic factors: M stage, N stage, Cystatin C, HCY, GGT, and age. These 6 features were confirmed to be consistent across all three algorithms, laying the foundation for subsequent nomogram development.

### Development of the prognostic nomogram

We used a multivariate Cox regression analysis to detect the independent association of these 6 features with prognosis. When incorporating these six features into the multivariate Cox regression analysis, the results indicated that all six features were independent factors affecting OS and PFS of CRC patients (**Tables 1**, **2**). Subsequently, we developed a prognostic nomogram based on these 6 hub clinical pathological to predict 1 to 5-year OS (**Figure 5A**) and PFS (**Figure 5B**). The nomogram showed that as the M/N stage progresses, and the levels of Cystatin C, Homocysteine, and Y-Glutamyl Transferase increase, along with age, the nomogram score increases, indicating a higher risk of poor prognosis. The calibration curves for 1-year, 3-year, and 5-year OS (**Supplementary Figures 1A–C**) and PFS (**Supplementary Figures 1D–F**) showed optimal consistency between predicted survival probabilities and actual observations. ROC curve results indicated that the OS nomogram had high prognostic prediction accuracy, with AUCs exceeding 75% for 1 to 5 years (**Supplementary Figure 2A**). Similarly, the PFS nomogram also had good prognostic prediction ability, with AUCs exceeding 77% for 1 to 5 years (**Supplementary Figure 2B**). Additionally, the C-index for the OS and PFS nomograms was 0.730 (95% CI: 0.707-0.753) and 0.722 (95% CI: 0.700-0.744), respectively. These results suggest that our prognostic nomogram based on these 6 clinical pathological features has good predictive accuracy for the prognosis of CRC patients. We also compared the accuracy of the nomogram with traditional pathological staging in predicting the prognosis of CRC patients using DCA. The results indicated that these nomograms were superior to traditional pathological staging in predicting the 1-year, 3-year, and 5-year prognosis of CRC patients (**Supplementary Figure 3**). According to the median nomogram score, we divided patients into high-score and low-score groups, finding that the OS and PFS of the high-score group were significantly lower than those of the low-score group (**Supplementary Figures 4A, B**).

**Table 1:** Key features of univariate and multivariate COX regression analysis for OS.

Clinicopathological characteristics	Univariate analysis	p value	Multivariate analysis	p value
M stage	7.302 (5.855,9.108)	<0.001	4.843 (3.825,6.132)	<0.001
N stage		<0.001		<0.001
N1	Ref		Ref	
N2	1.847 (1.501,2.273)	<0.001	1.666 (1.349,2.058)	<0.001
N3	4.099 (3.321,5.060)	<0.001	3.113 (2.475,3.915)	<0.001
Cystatin C	1.475 (1.229,1.771)	<0.001	1.453 (1.163,1.816)	0.001
Homocysteine	1.040 (1.024,1.056)	<0.001	1.025 (1.008,1.042)	0.004
γ-Glutamyl Transferase	1.005 (1.003,1.006)	<0.001	1.002 (1.000,1.003)	0.015
Age	1.009 (1.002,1.016)	0.017	1.008 (1.001,1.016)	0.023

**Table 2:** Key features of univariate and multivariate COX regression analysis for PFS.

Clinicopathological characteristics	Univariate analysis	p value	Multivariate analysis	p value
M stage	7.302 (5.855,9.108)	<0.001	4.733 (3.749,5.975)	<0.001
N stage		<0.001		<0.001
N1	Ref		Ref	
N2	1.847 (1.501,2.273)	<0.001	1.647 (1.343,2.020)	<0.001
N3	4.099 (3.321,5.060)	<0.001	3.121 (2.499,3.898)	<0.001
Cystatin C	1.475 (1.229,1.771)	<0.001	1.327 (1.055,1.671)	0.016
Homocysteine	1.040 (1.024,1.056)	<0.001	1.024 (1.008,1.041)	0.003
γ-Glutamyl Transferase	1.005 (1.003,1.006)	<0.001	1.002 (1.000,1.003)	0.023
Age	1.009 (1.002,1.016)	0.017	1.008 (1.001,1.015)	0.029

**Figure 5:**
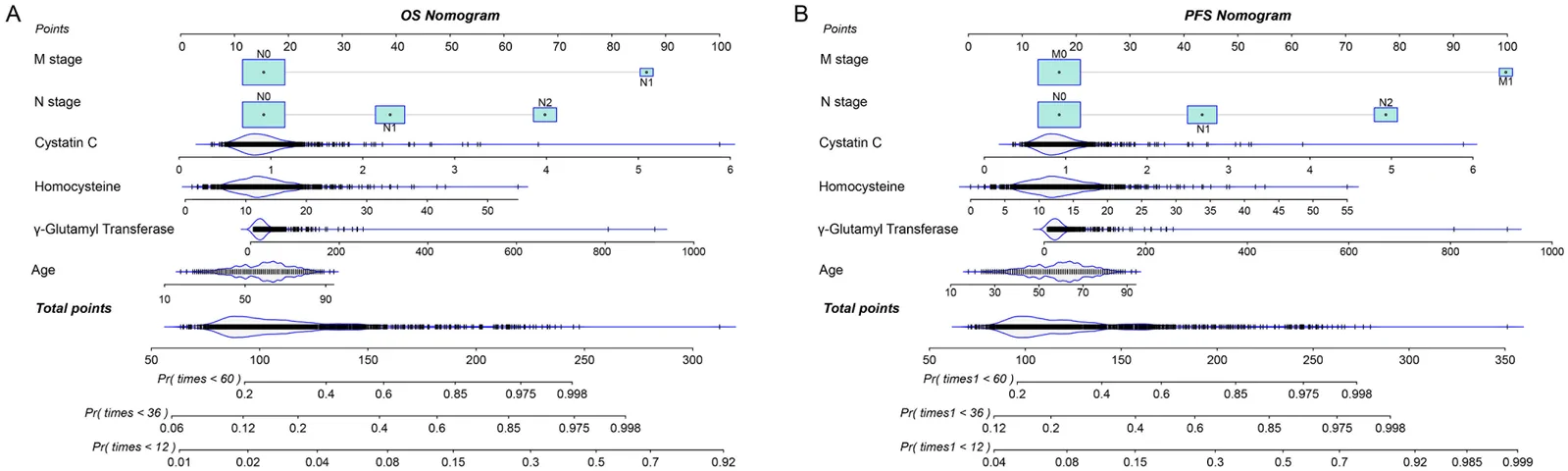
Prognostic nomograms for predicting overall survival (OS) and progression-free survival (PFS) in CRC patients. These nomograms were constructed based on the 6 hub prognostic features (M stage, N stage, Cystatin C, HCY, GGT, age) identified via multi-algorithm screening, and validated as independent prognostic factors by Cox regression. **(A)** OS nomogram: each feature is assigned a point value (top “Points” scale); for a given patient, points from individual features are summed to obtain a total score (bottom “Total points” scale), which corresponds to predicted 1-year, 3-year, and 5-year OS probabilities. **(B)** PFS nomogram: structured identically to the OS nomogram, it predicts 1-year, 3-year, and 5-year PFS probabilities. For both nomograms, higher total scores (e.g., advanced M/N stage, elevated Cystatin C/HCY/GGT, older age) correspond to higher risks of poor prognosis, providing a user-friendly tool for individualized survival prediction.

### Validation of the prognostic nomogram

We randomly divided the total population into two validation cohorts, A (632 cases) and B (631 cases), in a 1:1 ratio to validate the prognostic nomogram. There were no significant statistical differences in clinicopathological characteristics between the two cohorts (**Supplementary Table 4**). In both validation cohorts, calibration curves demonstrated good performance for the OS and PFS nomograms (**Supplementary Figures 5, 6**). ROC curves also indicated high accuracy of the OS and PFS nomograms in predicting prognosis at 1 year, 3 years, and 5 years (**Supplementary Figures 7, 8**). DCA results showed that the prognostic nomograms offered significant clinical benefits over traditional TNM staging (**Supplementary Figures 9, 10**). Patients with high scores had significantly worse prognosis compared to those with low scores (**Supplementary Figures 11, 12**). Furthermore, in validation cohort A, the C-index for the OS and PFS nomograms was 0.719 (95% CI: 0.689-0.749) and 0.717 (95% CI: 0.687-0.747), respectively. Similarly, in validation cohort B, the C-index for the OS and PFS nomograms was 0.743 (95% CI: 0.711-0.775) and 0.728 (95% CI: 0.697-0.759), respectively.

## Discussion

With the continuous development of artificial intelligence and machine learning technologies, an increasing number of medical fields are beginning to use these technologies to assist doctors in prognosis evaluation and treatment guidance (10, 11). Machine learning-based prognostic models for CRC have been widely explored, but existing studies often suffer from limitations such as single-algorithm bias, narrow feature scopes, or insufficient clinical utility validation—limiting their translational value. Against this background, our study innovatively addresses these gaps through methodological refinement, multi-dimensional feature integration, and rigorous clinical applicability verification, yielding findings with distinct novelty and translational value.

Most prior studies rely on a single machine learning algorithm for prognostic feature screening, which may introduce inherent bias due to algorithmic limitations. For example, a previous retrospective cohort study constructed a Random Forest-only model for long-term survival prediction of surgically treated CRC patients (12). However, the single-algorithm design could not exclude false-positive predictive features. Similarly, several studies adopted LASSO regression alone to screen clinical variables and construct OS nomograms, lacking cross-verification with other ensemble machine learning methods (13–15). In contrast, we integrated three complementary algorithms—LASSO regression (for regularization and linear feature prioritization), XGBoost (for ensemble learning and nonlinear relationship mining), and Random Forest (for decision-tree-based variable importance ranking)—and identified 6 hub prognostic features by taking the intersection of results from all three methods. This strategy avoids the overfitting or feature bias of single-algorithm approaches: LASSO reduces noise from 73 initial variables, XGBoost enhances generalization by combining weak learners, and Random Forest captures complex non-linear associations between factors and prognosis. The consistency of key features across algorithms (M stage, N stage, Cystatin C, Homocysteine, γ-Glutamyl Transferase, age) significantly improves the robustness of our feature set, laying a reliable foundation for subsequent model development. This multi-algorithm consensus screening strategy avoids the feature bias of single-algorithm approaches, and the consistent hub predictors identified across algorithms significantly improve the robustness of our final survival prediction model.

Existing CRC prognostic models typically focus on a narrow range of features, failing to capture the multi-factorial nature of prognosis. Our study is the first to comprehensively integrate pathological (M stage, N stage), serological (Cystatin C, Homocysteine, γ-Glutamyl Transferase), and demographic (age) factors into CRC prognostic nomograms—filling the gap of “single-type feature over-reliance” in existing research. Notably, the three serological markers we identified have synergistic prognostic value supported by prior literature but have never been combined with pathological and demographic factors in a single model. Cystatin C, a classic kidney function marker, has been linked to sarcopenia and tumor aggressiveness in CRC (16–18); our results confirm its independent association with poor OS/PFS, likely due to its reflection of systemic metabolic dysfunction and treatment tolerance. Homocysteine, an amino acid metabolite, is tied to inflammation and vascular damage. Our finding of its prognostic role aligns with recent work showing elevated levels correlate with CRC recurrence (19–21). γ-Glutamyl Transferase, a liver function enzyme, is associated with hepatic metastasis and treatment efficacy in CRC (22–24); its inclusion enhances the model’s ability to capture organ-specific tumor impact. Combined with M/N stage (pathological core predictors) and age (a proxy for comorbidity and immune senescence) (25), these features form a multi-dimensional panel that reflects tumor biology, systemic status, and patient characteristics—far more comprehensive than models relying on isolated feature types.

A critical limitation of many machine learning-based studies is the lack of explicit validation of clinical utility. Our study addresses this by conducting DCA, which quantifies net clinical benefit—an essential metric for translating models into practice. DCA results clearly showed that our nomograms outperform traditional TNM staging for 1/3/5-year OS/PFS predictions, meaning clinicians using our tool would achieve more accurate risk stratification without unnecessary overtreatment or undertreatment. Additionally, the features in our model are highly accessible in routine clinical practice: M/N stage is standard in pathological reporting, and the three serological markers are inexpensive, non-invasive, and widely available even in primary care settings (where most CRC patients are managed). This contrasts with models requiring specialized data, which are often limited to tertiary centers. The nomogram’s user-friendly visual design further enhances its applicability—clinicians can quickly calculate a patient’s total score and predict survival probabilities, guiding personalized treatment decisions (e.g., more frequent follow-up for high-score patients or de-escalation of therapy for low-score patients).

The results of this study have important implications for CRC clinical management. First, it demonstrates that multi-algorithm machine learning can uncover synergistic prognostic patterns across diverse feature types, providing a template for future studies aiming to develop robust predictive tools. Second, the integrated nomogram offers a “bridge” between complex data science and clinical practice—translating 73 variables into 6 actionable factors that improve upon traditional staging. Finally, our validation of serological markers (Cystatin C, Homocysteine, γ-Glutamyl Transferase) as independent prognostic factors suggests they could be incorporated into routine CRC follow-up panels to monitor disease progression.

However, this study also has limitations. First, it is a single-center retrospective study; while internal validation (1:1 cohort split) confirmed robust performance, multi-center external validation—including diverse ethnic populations—is needed to further verify generalizability. Second, we did not include genetic or molecular factors, which may contribute to CRC prognosis; future studies could integrate these with our current feature set to enhance model accuracy. Third, the interpretability of machine learning algorithms remains limited; exploring explainable AI (XAI) techniques (e.g., SHAP values) would help clinicians understand why a patient receives a high-risk score, improving trust in the model. Fourth, only the nomograms were statistically compared with traditional TNM staging, while no statistical comparison with other established prognostic models was conducted; we plan to address this gap in future research by benchmarking our model against previously validated machine learning-based CRC prognostic models. Fifth, all enrolled M1-stage patients were strictly limited to oligometastatic cases who received radical combined resection of primary tumor and limited metastases; patients with widespread unresectable metastases receiving only palliative primary resection were excluded during data screening. Even with this strict screening rule, inherent selection bias still exists in our retrospective single-center dataset. Large prospective clinical trials such as the iPACS trial and JCOG1007 have proven that primary tumor resection cannot bring survival benefits to CRC patients with extensive unresectable distant metastases. Therefore, our prognostic nomogram is not applicable to patients with widely metastatic disease. Further stratified multi-center research that separately analyzes oligometastatic and extensively metastatic M1 subgroups is necessary to expand the generalizable scope of our predictive model. Sixth, some metabolic indicators (cystatin C, homocysteine, γ-glutamyl transferase) are not routinely covered in basic biochemical test panels at primary medical institutions, which may restrict the widespread clinical promotion of our nomogram in grassroots hospitals lacking complete metabolic testing items.

## Conclusion

We have developed prognostic models using machine learning algorithms that integrate clinical, pathological, serological, and demographic factors to provide individualized predictions for the prognosis of CRC patients. These models outperform traditional TNM staging and serve as effective, user-friendly clinical tools for forecasting OS and PFS in CRC patients.

## Data Availability

The original contributions presented in the study are included in the article/**Supplementary Material**. Further inquiries can be directed to the corresponding authors.
