# Supplementary material for: Development of multi-algorithm machine learning models integrating novel serum biomarkers for survival prediction in colorectal cancer: a retrospective cohort study
Source: Front Oncol. 2026 Sep 15;16:1904591. doi: 10.3389/fonc.2026.1904591 (PMC13619412; doi:10.3389/fonc.2026.1904591)
Supplement: Supplementary file 1 [file Supplementaryfile1.docx]

**Fig S1.** Calibration curves of the prognostic nomograms for OS and PFS.

**Notes:** These calibration curves evaluate the agreement between nomogram-predicted survival probabilities and actual observed survival outcomes in the total CRC cohort (n=1263), a key metric for validating model reliability. (A–C) Calibration curves for 1-year (A), 3-year (B), and 5-year (C) OS: The x-axis represents the nomogram-predicted OS probability (%), and the y-axis represents the observed OS probability (%). (D–F) Calibration curves for 1-year (D), 3-year (E), and 5-year (F) PFS.

**Fig S2.** Time-dependent receiver operating characteristic (ROC) curves of the prognostic nomograms.

**Notes:** These curves assess the discriminative ability of the OS and PFS nomograms by quantifying their capacity to distinguish between patients with favorable vs. poor outcomes over 1–5 years. (A) ROC curves for OS: The x-axis represents the false positive rate (FPR, proportion of patients misclassified as high-risk), and the y-axis represents the true positive rate (TPR, proportion of patients correctly classified as high-risk). The area under the curve (AUC) for 1-year, 3-year, and 5-year OS is 0.751, 0.787, and 0.775, respectively—all >75%, indicating strong OS discriminative power. (B) ROC curves for PFS: AUC values for 1-year, 3-year, and 5-year PFS are 0.788, 0.782, and 0.772, respectively—all >77%, confirming the nomogram’s excellent ability to predict disease progression/recurrence.

**Fig S3.** Decision curve analysis (DCA) of the prognostic nomograms vs. traditional TNM staging.

**Notes:** This DCA quantifies the net clinical benefit of the nomograms compared to traditional TNM staging, addressing whether the model improves actionable clinical decision-making. (A) DCA for OS: The x-axis represents the threshold probability (clinician’s willingness to classify a patient as high-risk), and the y-axis represents net benefit (benefit of correct high-risk classification minus harm of incorrect classification). The nomogram curve lies above the TNM staging curve across most threshold probabilities, indicating higher net benefit. (B) DCA for PFS: The nomogram curve similarly outperforms TNM staging, confirming that using the nomogram reduces unnecessary overtreatment (for false positives) or undertreatment (for false negatives) compared to standard staging.

**Fig S4.** Kaplan-Meier survival curves of CRC patients stratified by median nomogram score

**Notes:** Patients were divided into high-score (≥ median score) and low-score (< median score) groups to validate the nomogram’s ability to stratify prognostic risk. (A) OS curves. (B) PFS curves.

**Fig S5.** Calibration curves of the prognostic nomograms in Validation Cohort A (n=632).

**Notes:** These curves validate the nomogram’s calibration in the first internal validation cohort (randomly split from the total population at 1:1), ensuring generalizability beyond the derivation cohort. (A) Calibration curves for 1–5-year OS: The x-axis represents predicted OS probability (%), and the y-axis represents observed OS probability (%). (B) Calibration curves for 1–5-year PFS: The x-axis represents predicted PFS probability (%), and the y-axis represents observed PFS probability (%).

**Fig S6.** Calibration curves of the prognostic nomograms in Validation Cohort B (n=631).

**Notes:** These curves further validate the nomogram’s calibration in the second internal validation cohort, ensuring robustness across independent subsets of the study population. (A) Calibration curves for 1–5-year OS: The x-axis represents predicted OS probability (%), and the y-axis represents observed OS probability (%). (B) Calibration curves for 1–5-year PFS: The x-axis represents predicted PFS probability (%), and the y-axis represents observed PFS probability (%).

**Fig S7.** Time-dependent ROC curves of the prognostic nomograms in Validation Cohort A (n=632).

**Notes:** These curves assess the nomogram’s discriminative ability in Validation Cohort A, ensuring its performance is not limited to the derivation cohort. (A) ROC curves for 1–5-year OS: AUC values are 0.685 (1-year), 0.761 (3-year), and 0.770 (5-year)—all >68%, confirming strong OS discriminative power in this cohort. (B) ROC curves for 1–5-year PFS: AUC values are 0.762 (1-year), 0.782 (3-year), and 0.772 (5-year)—all >76%, indicating excellent PFS discriminative ability, consistent with the derivation cohort.

**Fig S8.** Time-dependent ROC curves of the prognostic nomograms in Validation Cohort B (n=631).

**Notes:** These curves further validate the nomogram’s discriminative ability in Validation Cohort B, confirming consistent performance across independent subsets. (A) ROC curves for 1–5-year OS: AUC values exceed 74% (e.g., 1-year AUC = 0.78, 5-year AUC = 0.75), confirming strong OS discriminative power. (B) ROC curves for 1–5-year PFS: AUC values exceed 72% (e.g., 1-year AUC = 0.77, 5-year AUC = 0.73), indicating excellent PFS discriminative ability, aligning with results from the derivation and Validation Cohort A.

**Fig S9.** DCA of the prognostic nomograms vs. traditional TNM staging in Validation Cohort A (n=632).

**Notes:** This DCA validates the nomogram’s clinical benefit in Validation Cohort A, ensuring its utility extends beyond the derivation cohort. (A) DCA for OS: The nomogram curve outperforms TNM staging across most threshold probabilities, with higher net benefit. (B) DCA for PFS: The nomogram curve similarly lies above TNM staging, confirming that the model’s clinical utility is consistent in this validation cohort.

**Fig S10.** DCA of the prognostic nomograms vs. traditional TNM staging in Validation Cohort B (n=631).

**Notes:** This DCA further validates the nomogram’s clinical benefit in Validation Cohort B, reinforcing its translational value. (A) DCA for OS: The nomogram curve outperforms TNM staging across key threshold probabilities, with higher net benefit. (B) DCA for PFS: The nomogram curve maintains superior net benefit over TNM staging, confirming that the model improves clinical decision-making in independent patient subsets.

**Fig S11.** Kaplan-Meier survival curves of CRC patients in Validation Cohort A (n=632).

**Notes:** These curves validate the nomogram’s risk stratification ability in Validation Cohort A, ensuring it can distinguish between high- and low-risk patients in independent populations. (A) OS curves. (B) PFS curves.

**Fig S12**. Kaplan-Meier survival curves of CRC patients in Validation Cohort B (n=631).

**Notes:** These curves further validate the nomogram’s risk stratification ability in Validation Cohort B, confirming robust performance across all study subsets. (A) OS curves. (B) PFS curves.

**Table S1**. Complete List and Categorization of 73 Demographic and Clinical Data Items

| Categorization | Items |
| --- | --- |
| Demographic and anthropometric data | sex, age, height, weight, BMI |
| Tumor pathological data | T stage, N stage, M stage, perineural invasion, vascular invasion, pathological type, differentiation degree, tumor location, tumor size |
| Routine blood test data | white blood cell count, Red blood cell count, Hemoglobin, Platelet count, neutrophil count, lymphocyte count, monocyte count, eosinophils, basophils, mean corpuscular volume, mean RBC hemoglobin content, mean RBC hemoglobin concentration, RBC distribution width-CV, Mean platelet volume, platelet hematocrit, Platelet volume distribution width, hematocrit value |
| Biochemical and metabolic data | total bilirubin, direct bilirubin, indirect bilirubin, Direct/Total bilirubin ratio, total protein, prealbumin, albumin, globulin, Albumin/Globulin ratio, γ-Glutamyl Transferase, Total bile acid, Aspartate aminotransferase, Alanine aminotransferase, AST/ALT ratio, Alkaline phosphatase, Cholinesterase, urea, creatinine, uric acid, bicarbonate, cystatin C, potassium, sodium, calcium, Magnesium, total cholesterol, triglycerides, High-density lipoprotein cholesterol, Low-density lipoprotein cholesterol, homocysteine, Apolipoprotein_A, Apolipoprotein_B, Apolipoprotein_AI, Lipoprotein a, blood glucose, Activate partial thrombin time |
| Tumor marker data | CEA |
| Treatment and comorbidity data | radiotherapy, chemotherapy, hypertension, diabetes, complication |

**Table S2** Clinicopathological characteristics of patients with colorectal cancer.

| Clinicopathological characteristics | Overall |
| --- | --- |
|  | n=1263 |
| Sex |  |
| Man | 799 (63.3) |
| Woman | 464 (36.7) |
| Age (mean (SD)) | 59.23 (12.63) |
| BMI (median [IQR]) | 22.07 (20.00, 24.44) |
| Hypertension (Yes) | 221 (17.5) |
| Diabetes (Yes) | 84 ( 6.7) |
| T stage |  |
| T1 | 44 ( 3.5) |
| T2 | 274 (21.7) |
| T3 | 677 (53.6) |
| T4 | 268 (21.2) |
| N stage |  |
| N0 | 710 (56.2) |
| N1 | 342 (27.1) |
| N2 | 211 (16.7) |
| M stage |  |
| M0 | 1152 (91.2) |
| M1 | 111 (8.8) |
| TNM stage |  |
| I | 252 (20.0) |
| II | 427 (33.8) |
| III | 473 (37.5) |
| IV | 111 (8.8) |
| Perineural invasion (Yes) | 128 (10.1) |
| Vascular invasion (Yes) | 218 (17.3) |
| Differentiation (Poor) | 165 (13.1) |
| Location (Rectal cancer) | 646 (51.1) |
| Tumor size (median [IQR]) | 4.50 (3.50, 6.00) |
| Radiotherapy (Yes) | 107 ( 8.5) |
| Chemotherapy (Yes) | 562 (44.5) |
| Recurrence (Yes) | 348 (27.6) |
| Length of stay (median [IQR]) | 17.00 (11.00, 21.00) |
| Hospitalization cost (median [IQR]) | 49828.44 [44810.71, 56390.06] |
| CEA (median [IQR]) | 3.94 (2.12, 11.44) |

**Table Note:** CRC, colorectal cancer; BMI, body mass index.

**Table S3** Clinicopathological characteristics between alive and death.

| Clinicopathological characteristics | Overall survival | | p |
| --- | --- | --- | --- |
|  | Alive (n=745) | Death (n=518) |  |
| Sex |  |  | 0.831 |
| Man | 469 (63.0) | 330 (63.7) |  |
| Woman | 276 (37.0) | 188 (36.3) |  |
| Age (mean (SD)) | 58.56 (12.02) | 60.19 (13.41) | 0.024 |
| BMI (median [IQR]) | 22.37 (20.13, 24.61) | 21.78 (19.72, 24.03) | 0.004 |
| Hypertension (Yes) | 120 (16.1) | 101 (19.5) | 0.138 |
| Diabetes (Yes) | 45 (6.0) | 39 (7.5) | 0.353 |
| T stage |  |  | <0.001 |
| T1 | 36 (4.8) | 8 (1.5) |  |
| T2 | 212 (28.5) | 62 (12.0) |  |
| T3 | 375 (50.3) | 302 (58.3) |  |
| T4 | 122 (16.4) | 146 (28.2) |  |
| N stage |  |  |  |
| N0 | 507 (68.1) | 203 (39.2) | <0.001 |
| N1 | 182 (24.4) | 160 (30.9) |  |
| N2 | 56 (7.5) | 155 (29.9) |  |
| M stage |  |  | <0.001 |
| M0 | 743 (9.7) | 409 (79.0) |  |
| M1 | 2 (0.3) | 109 (21.0) |  |
| TNM stage |  |  | <0.001 |
| I | 203 (27.2) | 49 (9.5) |  |
| II | 304 (40.8) | 123 (23.7) |  |
| III | 236 (31.7) | 237 (45.8) |  |
| IV | 2 (0.3) | 109 (21.0) |  |
| Perineural invasion (Yes) | 53 (7.1) | 75 (14.5) | <0.001 |
| Vascular invasion (Yes) | 86 (11.5) | 132 (25.5) | <0.001 |
| Differentiation (Poor) | 78 (10.5) | 87 (16.8) | 0.001 |
| Location (Rectal cancer) | 370 (49.7) | 276 (53.3) | 0.227 |
| Tumor size (median [IQR]) | 4.20 (3.50, 5.50) | 5.00 (3.50, 6.00) | 0.001 |
| Radiotherapy (Yes) | 63 (8.5) | 44 (8.5) | 0.999 |
| Chemotherapy (Yes) | 321 (43.1) | 241 (46.5) | 0.249 |
| Recurrence (Yes) | 24 (3.2) | 324 (62.5) | <0.001 |
| Length of stay (median [IQR]) | 15.00 (10.00, 20.00) | 19.00 (14.00, 23.00) | <0.001 |
| Hospitalization cost (median [IQR]) | 48694.05 (44497.64, 54923.81) | 51636.26 (45503.97, 59462.03) | <0.001 |
| CEA (median [IQR]) | 3.19 (1.74, 7.06) | 6.44 (2.74, 23.11) | <0.001 |

Table Note: CRC, colorectal cancer; BMI, body mass index.

**Table S4** Clinicopathological characteristics between validation cohort A and validation cohort B.

| Clinicopathological characteristics | Validation cohort A (n=632) | Validation cohort B (n=631) | p |
| --- | --- | --- | --- |
| Sex |  |  | 0.667 |
| Man | 404 (63.9) | 395 (62.6) |  |
| Woman | 228 (36.1) | 236 (37.4) |  |
| Age (mean (SD)) | 59.02 (12.89) | 59.43 (12.37) | 0.559 |
| BMI (median [IQR]) | 22.07 (19.92, 24.47) | 22.07 (20.08, 24.22) | 0.888 |
| Hypertension (Yes) | 116 (18.4) | 105 (16.6) | 0.467 |
| Diabetes (Yes) | 35 ( 5.5) | 49 ( 7.8) | 0.14 |
| T stage |  |  |  |
| T1 | 22 ( 3.5) | 22 ( 3.5) | 0.657 |
| T2 | 146 (23.1) | 128 (20.3) |  |
| T3 | 330 (52.2) | 347 (55.0) |  |
| T4 | 134 (21.2) | 134 (21.2) |  |
| N stage |  |  |  |
| N0 | 352 (55.7) | 358 (56.7) | 0.715 |
| N1 | 169 (26.7) | 173 (27.4) |  |
| N2 | 111 (17.6) | 100 (15.8) |  |
| M stage |  |  | 0.114 |
| M0 | 64 (10.1) | 47 (7.4) |  |
| M1 | 568 (89.9) | 584 (92.6) |  |
| TNM stage |  |  |  |
| I | 132 (20.9) | 120 (19.0) | 0.215 |
| II | 202 (32.0) | 225 (35.7) |  |
| III | 234 (37.0) | 239 (37.9) |  |
| IV | 64 (10.1) | 47 ( 7.4) |  |
| Perineural invasion (Yes) | 60 ( 9.5) | 68 (10.8) | 0.508 |
| Vascular invasion (Yes) | 106 (16.8) | 112 (17.7) | 0.7 |
| Differentiation (Poor) | 85 (13.4) | 80 (12.7) | 0.747 |
| Location (Rectal cancer) | 324 (51.3) | 322 (51.0) | 0.978 |
| Tumor size (median [IQR]) | 4.50 (3.50, 6.00) | 4.50 (3.50, 5.50) | 0.217 |
| Radiotherapy (Yes) | 56 ( 8.9) | 51 ( 8.1) | 0.692 |
| Chemotherapy (Yes) | 284 (44.9) | 278 (44.1) | 0.796 |
| Recurrence (Yes) | 191 (30.2) | 157 (24.9) | 0.039 |
| Length of stay (median [IQR]) | 17.00 (11.00, 21.00) | 16.00 (11.00, 20.50) | 0.309 |
| Hospitalization cost (median [IQR]) | 50449.07 (45101.79, 56877.42) | 49445.87 (44551.74, 55726.85) | 0.228 |
| CEA (median [IQR]) | 4.04 (2.16, 12.48) | 3.90 (2.05, 9.94) | 0.366 |

Table Note: CRC, colorectal cancer; BMI, body mass index.
